# Computational Discovery of Putative Leads for Drug Repositioning through Drug-Target Interaction Prediction

**DOI:** 10.1371/journal.pcbi.1005219

**Published:** 2016-11-28

**Authors:** Edgar D. Coelho, Joel P. Arrais, José Luís Oliveira

**Affiliations:** 1 Department of Electronics, Telecommunications and Informatics (DETI), Institute of Electronics and Telematics Engineering of Aveiro (IEETA), University of Aveiro, Aveiro, Portugal; 2 Department of Informatics Engineering (DEI), Centre for Informatics and Systems of the University of Coimbra (CISUC), University of Coimbra, Coimbra, Portugal; University of Houston, UNITED STATES

## Abstract

*De novo* experimental drug discovery is an expensive and time-consuming task. It requires the identification of drug-target interactions (DTIs) towards targets of biological interest, either to inhibit or enhance a specific molecular function. Dedicated computational models for protein simulation and DTI prediction are crucial for speed and to reduce the costs associated with DTI identification. In this paper we present a computational pipeline that enables the discovery of putative leads for drug repositioning that can be applied to any microbial proteome, as long as the interactome of interest is at least partially known. Network metrics calculated for the interactome of the bacterial organism of interest were used to identify putative drug-targets. Then, a random forest classification model for DTI prediction was constructed using known DTI data from publicly available databases, resulting in an area under the ROC curve of 0.91 for classification of out-of-sampling data. A drug-target network was created by combining 3,081 unique ligands and the expected ten best drug targets. This network was used to predict new DTIs and to calculate the probability of the positive class, allowing the scoring of the predicted instances. Molecular docking experiments were performed on the best scoring DTI pairs and the results were compared with those of the same ligands with their original targets. The results obtained suggest that the proposed pipeline can be used in the identification of new leads for drug repositioning. The proposed classification model is available at http://bioinformatics.ua.pt/software/dtipred/.

## Author Summary

The emergence of multi-resistant bacterial strains and the existing void in the discovery and development of new classes of antibiotics is a growing concern. Indeed, some bacterial strains are now resistant to last-line antibiotics and considered untreatable. Drug repositioning has been suggested as a strategy to minimize time and cost expenses until the drug reaches the market, compared to traditional drug design. Drug-target interactions (DTIs) are the basis of rational drug design and thus, we proposed a computational approach to predict DTIs solely based on the primary sequence of the protein and the simplified molecular-input line-entry system of the ligand. In addition, network metrics are used to identify vital putative drug-targets in bacteria. Molecular docking experiments were performed to compare the binding affinities between a given ligand and a putative drug-target, as well as with their original targets. According to the docking results, the predicted DTIs have better or similar binding activities than the ligand and their real target, indicating the validity of the proposed model.

## Introduction

Antibacterial resistance is becoming more frequent and is a growing concern, as bacterial resistance to last-line antibiotics has been steadily increasing and is already high globally [1,2]. Development of antibacterial resistance is the result of a cascade of events triggered by continued selective pressure of routinely used antibiotics, constituting a major medical and pharmaceutical challenge. In response to continued selective pressure the bacterial genome undergoes rapid evolution, which in turn is accelerated by the heavy focus on the same microbial pathways (protein synthesis, nucleic acid synthesis, cell wall synthesis and folate synthesis) [3,4]. Today, more than ever, new antibiotics or prodrugs able to neutralize antimicrobial resistant pathogens are necessary.

A growing strategy in drug screening for the past decade is drug repositioning, or repurposing. By focusing on one of the undesired effects of an already commercialized drug in an attempt to make it the main effect, it is possible to reposition that drug for new uses [5]. This strategy can greatly reduce the cost of lead screening and the time required for a drug to reach the market [6,7]. Some examples of successfully repositioned drugs for uses different from their original indications include bupropion, fluoxetine, thalidomide and sildenafil [8,9]. Sildenafil is probably the most popular example, which was initially used to treat hypertension, then angina, and currently for erectile dysfunction [10]. However, the repurposing of thalidomide should not be taken lightly, as it is an example of a withdrawn drug that could be reintroduced in the market [11]. From another perspective, neglected and rare diseases are also becoming increasingly attractive for pharmaceutical companies, which can be partially attributed to the smaller initial investment necessary to repurpose drugs for such diseases [9].

This was on the basis of the proposed works by Cheng *et al*. [12] and Yang *et al*. [13]. Yang *et al*. [13] developed the conditional random field (CRF) method, which integrates genomic, chemical, functional and pharmacological data, in addition to the topology of DTI networks. The CRF is a probabilistic graph model able to encode the drug-target network for DTI prediction. They apply a stochastic gradient ascent approach and the contrastive divergence algorithm to train their model and to identify the hidden associations between drugs and targets [13]. While this methodology may have the potential to be applied to reposition certain drugs, the use of functional similarity dismisses its use for the drug repositioning in infectious diseases. The most likely result of using functional similarity for this purpose would be the prediction of an antibacterial drug that would continue the selective pressure in the target microorganism, perpetuating antibacterial resistance.

The work by Cheng *et al*. [12] consisted in the construction of a bipartite graph of drugs approved by the United States Food and Drug Administration linked by binary associations to their respective protein targets to infer drug-target interactions (DTI). Their proposed method, network-based inference (NBI), uses known drug-target bipartite network topology similarity to predict unknown DTI. NBI considers transition processes over the bipartite graph, and thus, if a drug and a protein target interact, it is possible to compute the predictive score. The predictive score is calculated based on the number of drugs associated with each target, and on the degree of these drugs. Some of their predictions were validated by *in vitro* assays, confirming that five drugs had pharmacological effects on alternative targets [12]. While these results are very promising for drug repurposing, we believe their proposed methodology could not be used to solve the problem present here. Considering that Cheng *et al*. [12] used a DTI network composed of human protein targets and their respective drugs to construct their inference model, if they used a DTI network of a bacterial species of interest and the drugs targeting it (*i*.*e*., antibiotic drugs), the selective pressure problem would persist and result in antibacterial resistance.

Drug repositioning is especially challenging in infectious diseases for a number of reasons. First, most antibiotics were originally isolated by screening soil-derived *Actinomycetes* between 1940 and 1960 [14]. Shortly after, the productivity of this antibiotic discovery strategy started decaying rapidly, becoming obsolete. Second, the antibacterial “spectrum expansion” methodology, which consists of testing a drug able to suppress one bacterium species against other species [15], is too expensive and time-consuming. In addition, despite high-throughput screening against defined targets and rational drug design yielding several compounds, the compounds identified were not effective at penetrating bacterial cells [14]. Nonetheless, efforts to reposition drugs for infectious diseases are becoming increasingly attractive, especially those using computational methodologies [16,17]. Computational methodologies allow rapid and inexpensive screening of a broad spectrum of drugs and targets, either by screening ligands for a certain drug-target, or screening potential drug-targets for a specific ligand.

Nzila *et al*. [15] reviewed several strategies used to reposition drugs for the treatment of multi- and drug-resistant malaria and tuberculosis. Five strategies for drug repositioning were presented: 1) assess similarity in cell biology and biological processes, using compounds that target pathways that also exist in the microorganisms responsible for malaria and tuberculosis; 2) explore the microorganism genome information, aiming to identify putative drug targets already validated in another organism; 3) revisit data from failed drug reposition attempts, as inherent variables (e.g., animal model chosen, toxicity) could be poorly chosen or dealt with; 4) observe co-infection drug treatment efficacy thoroughly, as many diseases occur as co-infections with malaria and tuberculosis, and; 5) screen old and existing drugs. Indeed, in a recent approach Iwata *et al*. [16] proposed a statistical model to infer new drug-disease associations based on known drug-disease interaction knowledge. In their approach, each drug-disease pair was defined a descriptor based on the phenotypic effects of drugs (*e*.*g*., main effect and side effects) and with various molecular features of diseases (*e*.*g*., disease-causing genes, diagnostic markers, disease-related pathways, and environmental factors). Berenstein *et al*. [17] took advantage of extensively studied organisms to develop an integrative network model for the identification of bioactive drug-like molecules and candidate drug targets in neglected pathogen proteomes. More recently, Savoia [18] reviewed several promising experimental studies on drug repurposing of existing drugs for infectious diseases, most of them identified serendipitously or by exploring the side-effects of the drugs.

Computational drug repurposing approaches invariably make use of previously known drug-target associations. Finding alternative targets for known drugs has the added benefit of advancing into clinical trials sooner, as their pharmacokinetics and safety profiles are known by the regulatory authorities [19].

In this paper, we propose a methodology for screening putative DTIs for drug repositioning. The proposed pipeline allows the identification of potential drug-targets in any bacterial species of interest, and the prediction of putative DTIs between the identified drug-targets and already commercialized drugs. The newly identified DTIs can provide key leads for drug repurposing towards problematic pathogens, being a time and cost-effective strategy to support the development of new antibacterials.

## Results and Discussion

### Classification model performance assessment and comparison

We constructed our random forest classification model based on the training set, and using the values found by grid search for *n_estimators* (number of trees in the forest) and *max_features* (number of features to consider when looking for the best split). We performed five-fold cross-validation (internal validation) and tested the classification model against data sets independent of the training data (external validation) to evaluate classification performance. The AUCs for five-fold cross-validation and external data set validation were 0.99 and 0.91, respectively. After classifying the external validation data set we computed the confusion matrix for the predicted instances (Table 1). These results indicate that the presented model is valid and able to classify unseen data.

**Table 1:** Confusion matrix of external validation data set classification.

	Predicted positive	Predicted negative
Condition positive	2,792 (TP)	542 (FN)
Condition negative	69 (FP)	5,126 (TN)

TN–true negative; FP–false positive; FN–false negative; TP–true positive

### Analysis of the impact of network metrics

The network metrics calculated for the proteins in the methicillin-resistant *S*. *aureus* (strain COL–MRSA COL) interactome were sorted by their betweenness centrality (BC) values in descending order and filtered for a subgraph centrality (SC) value greater than 1023 as the best putative drug-targets. Table 2 lists the ten best scoring proteins according to the calculated network metrics. The prokaryotic DNA-directed RNA polymerase is an enzyme with multiple subunits responsible for transcription in bacteria. It is an appealing drug target due to its essentiality for bacterial growth and survival and its different features from mammalian counterparts [20,21]. According to DrugBank, Rifabutin targets both the alpha and beta subunits in Escherichia coli strain K12, while the beta subunit is targeted by Rifapentine (in *Mycobacterium tubercul*osis), Rifampicin, Rifaximin, and Rifalazil (in *E*. *coli* strain K12).

**Table 2:** Top ten best putative drug-targets.

STRING ID	UniProt ID	Protein name	SC	BC
SACOL2213	Q5HDY4	DNA-directed RNA polymerase subunit alpha	1.85E+23	0.0329
SACOL0591	Q5HID0	30S ribosomal protein S12	2.76E+23	0.0198
SACOL0588	Q5HID3	DNA-directed RNA polymerase subunit beta	1.17E+23	0.0178
SACOL2675	Q5HCP4	Accessory Sec system protein translocase subunit SecY2	1.01E+23	0.0128
SACOL1292	Q5HGF8	30S ribosomal protein S15	2.65E+23	0.0112
SACOL0593	Q5HIC8	Elongation factor G	2.82E+23	0.0093
SACOL2234	Q5HDW3	50S ribosomal protein L22	3.29E+23	0.0049
SACOL2233	Q5HDW4	30S ribosomal protein S3	3.11E+23	0.0047
SACOL2207	Q5HDZ0	50S ribosomal protein L13	2.94E+23	0.0046
SACOL0545	Q5HIH4	50S ribosomal protein L25	1.06E+23	0.0045

SC–Subgraph centrality; BC–Betweenness centrality

The ribosome is responsible for protein synthesis in the cell and is composed of two subunits, the 50S (larger) and 30S (smaller). Drugs that target ribosomal proteins to inhibit bacterial protein synthesis are either 50S inhibitors (chloramphenicol, clindamycin, macrolides, and pleuromutilins) or 30S inhibitors (tetracycline and aminoglycosides) [22–24].

The movement of tRNA and mRNA through the ribosome at the end of each round of polypeptide elongation is catalyzed by the prokaryotic elongation factor G (EF-G) [25]. Fusidic acid inhibits ribosomal peptide elongation (and ribosome recycling) by targeting EF-G, forming a strong complex when EF-G is ribosome-bound [26].

Protein secretion is crucial to export virulence factors and thus, to improve pathogenic survivability. The accessory Sec system is a specialized export system found in mycobacteria and some Gram-positive bacteria, where the common element is the accessory SecA protein SecA2. It was reported that in the specific case of S. aureus the SecA2/SecY2 system is required for the export of the serine-rich surface protein adhesion (SraP), an important virulence determinant in endovascular infection [27,28]. Inhibition of SecY2 was found to prevent SraP surface expression almost completely [28].

These literature findings suggest this heuristic is a good predictor to identify putative drug-targets in bacterial species of interest. To generate our test data set we combined the best ten proteins with the 3,081 unique ligands in our training and test data sets in an all-against-all fashion, resulting in 30,810 DTIs in the test set.

### Analysis of predicted putative drug-target interactions

In our model we opted to predict the probability of each DTI pair to interact, i.e., the probability of a given DTI pair to be classified as a positive interaction. On a random forest classifier the predicted class probability is computed as the mean probability of the predicted class from the trees in the forest, where the single tree class probability is the fraction of samples of the same class in a leaf. This allowed us to sort DTI pairs by their class probabilities for easier identification of the most probable putative DTIs. We selected the five most probable DTIs according to our classification model for further analysis (Table 3). According to UniProt, the proteins involved in these DTIs did not have solved tertiary structures at the time of writing. Thus, we performed ab initio homology modeling following a well-established strategy [29]. First, we used the I-TASSER [30–32] online server to predict the tertiary structure of the proteins with UniProt IDs Q5HIC8, Q5HID3, and Q5HCP4, using the default parameters. The I-TASSER server uses three metrics to measure the confidence of each generated model: 1) C-score, which is calculated based on the significance of threading template alignments and the convergence parameters of the structure assembly simulations; 2) RMSD, which is an average distance of all residue pairs in two structures, and; 3) TM-score, which weighs the small distance between all residue pairs stronger than the big distance, making the score insensitive to the local modeling error (disregarded in RMSD). However, RMSD and TM-score are used when the native structure is known, meaning their values in I-TASSER are predicted based on the C-score. The C-score, RMSD and TM-score values for Q5HIC8 are 1.53, 4.8±3.1Å, and 0.93±0.06, respectively. For Q5HID3 these values are 0.19, 8.8±4.6Å, and 0.74±0.11. Lastly, for Q5HCP4, the C-score is 1.20, RMSD is 4.3±2.9Å, and TM-score is 0.88±0.07. In general, C-score values are comprised between -5 and 2, with 2 being a good indicator of model confidence. Since the C-score of Q5HID3 fell short of those of Q5HIC8 and Q5HCP4 we decided to discard the modeled tertiary structure.

**Table 3:** Five best scoring putative drug-target interactions.

UniProt ID	Protein Name	ZINC ID	Ligand name	Class probability
Q5HIC8	Elongation factor G	ZINC85537089	Proglumetacin maleate	0.93
Q5HID3	DNA-directed RNA polymerase subunit beta	ZINC01550477	Lapatinib	0.93
Q5HID3	DNA-directed RNA polymerase subunit beta	ZINC85537027	Tacrolimus	0.92
Q5HCP4	Accessory Sec system protein translocase subunit SecY2	ZINC19418959	Trifluoperazine dihydrochloride	0.92
Q5HIC8	Elongation factor G	ZINC01535101	Rosuvastatin calcium	0.91

The accuracy of the generated models was estimated using ProSA-web [33], an established tool used for the refinement and validation of experimental protein structures and in structure prediction and modeling.

This tool parses the coordinates of the structure and evaluates its energy using a distance-based pair potential and a potential capable of detecting solvent exposed residues. The Z-score, an indicator of overall model quality, is calculated using these energies. Specifically, it measures the deviation of the total energy of the model’s structure, considering an energy distribution derived from random conformations [33]. All possible conformations of a given protein have associated energy values. The number of conformations per energy interval, that is, the energy density *N(E)*, characterizes the energy distribution of said protein. By the law of large numbers one can assume that the energy density follows a Gaussian distribution, defined by the average energy *Ē*, and standard deviation *σ*. Since every distribution has an average and a standard deviation, it is possible to normalize energy values, even without knowing the shape of this distribution. Thus,

$$E\to \frac{\left(E-Ē\right)}{\sigma }\approx z$$ (1)

**Fig 1:**
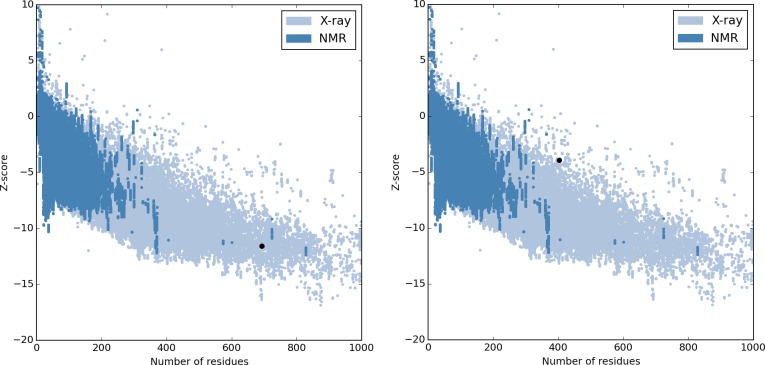
ProSA-web overall model quality output for Q5HIC8 (left) and Q5HCP4 (right), respectively. Panels show these proteins are within the range of scores typically found for proteins of similar size.

These normalized values are called z-scores [34]. Lower z-score values are correlated with typical native structures of similar size, while z-scores outside the characteristic range of the native proteins indicate erroneous structures [33]. Z-score values for Q5HIC8 and Q5HCP4 were -10.39 and -3.95, respectively, suggesting the quality of the modeled tertiary structures (Fig 1).

Following ab initio structural modeling and validation, we performed docking experiments between our predicted DTIs to test the theoretical viability of the ligands to actually bind the modeled proteins. In addition, we also performed docking experiments between these ligands and their real targets to create a benchmark. All docking experiments were carried out using the SwissDock web-server [35] and AutoDock4 [36]. Table 4 summarizes the docking experiments and benchmarks performed, as well as their results. The results of SwissDock docking suggest that the ligands ZINC85537089 and ZINC01535101 have a greater binding affinity to Q5HIC8 than to the targets they were originally synthesized for. Although not possessing the lowest full fitness, the Q5HPC4-ZINC19418959 DTI pair still has a higher binding affinity than the P26447-ZINC19418959 pair. Trifluoperazine dihydrochloride was shown to have antiplasmid effects on a range of bacterial species [37,38]. Furthermore, it was reported that Prochlorperazine (ZINC19796018), an antipsychotic drug with MCS Tanimoto similarity of 0.8276 with Trifluoperazine dihydrochloride (ZINC19418959), also possesses antibacterial activity against several species [39]. Similar studies show evidence that statins, including Rosuvastatin calcium (ZINC01535101), also present activity against a range of bacterial species [40,41]. Proglumetacin maleate (ZINC85537089) belongs to the acetic acid derivatives and related substances class. While acetic acid has known antibacterial properties [42,43], Proglumetacin maleate does not have any antibacterial effects and had been actually labeled as non-antibacterial [44,45].

**Table 4:** Results of the molecular docking experiments performed for predicted and real (benchmark) DTIs.

ZINC ID	UniProt ID	Target type	PDB ID	SD	AD4
85537089	Q5HIC8	Predicted	N/A	-2.69	-2.03
	P23219	Real	1CQE	-2.06	-3.93
	P35354	Real	5F19	-2.29	-3.98
19418959	Q5HCP4	Predicted	N/A	-0.89	-5.69
	P63316	Real	1J1D	-1.50	-5.28
	P62158	Real	1CLL	-1.29	-7.16
	P26447	Real	2Q91	-0.59	-6.83
	P14416	Real	5AER	-1.04	-5.56
01535101	Q5HIC8	Predicted	N/A	-2.90	-1.59
	P04035	Real	1DQ8	-2.20	-2.63

Values are presented in cal/mol. SD–SwissDock; AD4 –AutoDock4.

The results of AutoDock4 docking were not so optimistic, with only ZINC19418959 showing greater binding affinity to the predicted protein Q5HCP4 than to two of its real targets (P63316 and P14416). Nonetheless, the evidence shown here is highly suggestive that the identified compounds are able to bind to the predicted drug-targets, attesting the performance of the proposed methodology. Indeed, we looked further into the antimicrobial activity of acetic acid and found reports of its ability to directly eradicate mature biofilms [46] and inhibit oral microorganisms [47].

Experimental testing will be decisive in validating the presented findings. Namely, if these DTIs actually occur, the identified ligands may not be able to cross the cell wall and cell membrane, which would most likely require lead optimization to improve selectivity to the target and efficiency of the ligand. Still, the robustness and reliability of the proposed pipeline can be attested, as it performed well in both internal validation and external validation data sets.

Overall, we show that the combined use of network metrics, namely subgraph centrality and betweenness centrality, are extremely useful for finding potential drug-targets in MRSA. Most of the ten best putative drug targets were part of the already heavy focused microbial pathways (protein synthesis, nucleic acid synthesis and cell wall synthesis), which demonstrates that this heuristic is able to identify essential proteins. Considering this, identification of the accessory Sec system protein translocase subunit SecY2 as a putative drug-target seems especially relevant, as it is part of a pathway that has not received much attention for antibacterial development. Future studies will focus on this and other less explored pathways for antimicrobial development.

Even though we used the MRSA interactome as a case-study, this pipeline was developed to be applied to any pathogen species of interest, as long as their interactome is at least partially known. By reducing the number of possible drug-targets it is possible to save time and funds to be directed to investigating the shorter drug-target pool. Moreover, DTI prediction further narrows the lead screening window, allowing the possibility of drug-repurposing. Finally, since these drugs are already commercialized there should be no inherent risks in using them as antibacterials.

## Methods

### Pipeline overview

**Fig 2:**
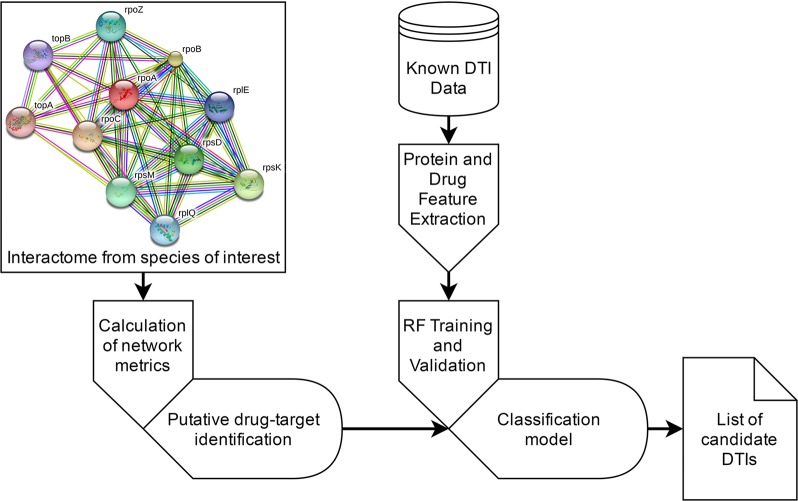
Diagram of the proposed pipeline.

The proposed approach is schematized in Fig 2. Known DTI data were collected from publicly available databases. From the ligand’s simplified molecular-input line-entry system (SMILE) representation of a ligand the chemical structure data and physicochemical descriptors are retrieved and encoded. Similarly, from the primary sequence of a protein a variety of physicochemical descriptors are retrieved. These descriptors are used to generate the feature vectors that represent DTI pairs.

The proposed classification model uses random forest (RF) [48], as these run efficiently on large data sets, provide accurate estimates, are able to estimate the most important features in the classification task, and are less prone to overfitting. Classifier validation includes internal (five-fold cross-validation) and external validation on an independent data set. The classification model is then used to predict putative DTIs between the estimated crucial drug-targets in the methicillin-resistant *Staphylococcus aureus* (strain COL–UniProt taxonomy ID: 93062) and all the drugs in our training and test data sets. The most essential drug-targets were estimated by a combination of subgraph centrality (SC) and the betweenness centrality (BC) of each protein in the bacterial interactome, as SC is highly correlated with the lethality of individual proteins removed from the proteome, and BC is likely to be associated with protein essentiality [49,50]. The methicillin-resistant *S*. *aureus* COL (MRSA) interactome was used to test and validate the proposed pipeline. Finally, we use the SwissDock server and AutoDock4 to perform docking simulations for the best scoring predicted DTIs. The docking process for SwissDock was set to “Accurate” and the region of interest was set to default, as this docking is flexible. For AutoDock4, the search parameters were set to long (25,000,000 evaluations) and carried out by a Genetic Algorithm. The docking process was performed using a Lamarckian Genetic Algorithm. Hydrogen was added to each protein undergoing docking testing and Gasteiger charges were assigned. The spacing between grid points used was the default value (0.375 Å). Additional docking simulations between the same ligands and their original drug-targets are performed to establish benchmarks for comparison.

SwissDock is based in the EADock DSS engine [35,51]. In this engine, binding models are generated and scored using a simple fitness function, minimized, and then clustered and evaluated according to their full fitness [51]. The most stable DTI complexes are those with the lowest docking score values. Since the SwissDock server [35] allows the direct upload of PDB codes (for target selection) and ZINC database accession identifiers (for ligand selection), pre-processing of the structures for the docking experiments by us was not required. Instead, this process is automatically performed in the SwissDock server, where the input molecules are converted to the CHARMM [52] format. This is the selected format since docking assays are performed in the CHARMM22/27 all-hydrogen force field. Protein target and ligand setup are thoroughly described in [35]. The EADock DSS engine generates between 5,000 and 15,000 binding models near the target cavities of the entire protein surface and simultaneously estimates their CHARMM energies on a grid. Binding models with the most favorable energies are then ranked, considering the solvent effect using the FACTS implicit solvation model [53]. The most favorable binding model clusters are then presented in the results file.

### Positive data set construction

**Fig 3:**
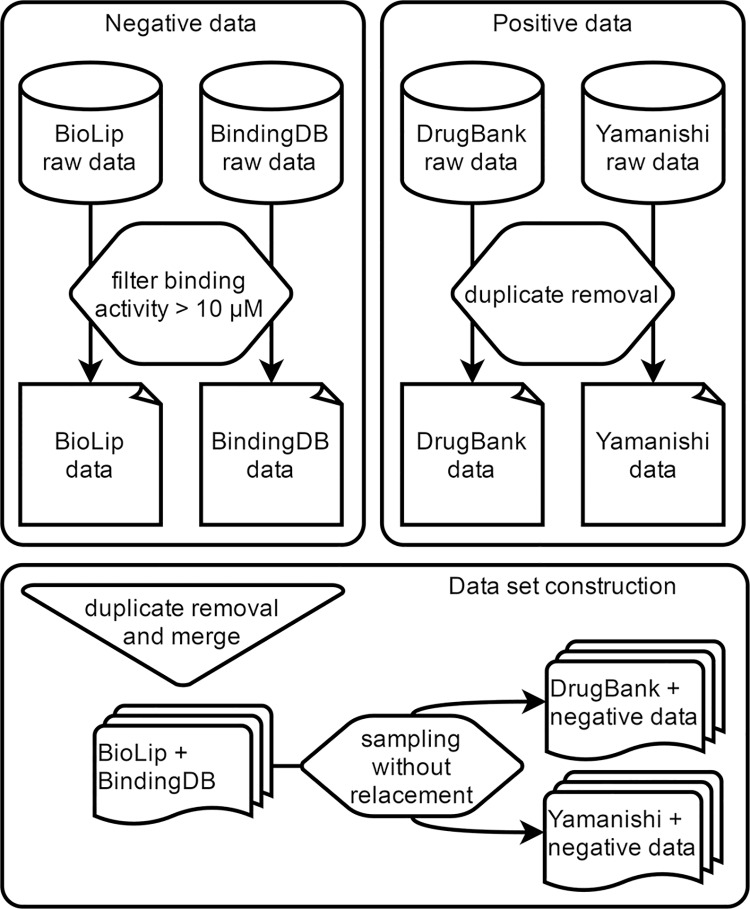
Data set construction.

In this work we collected positive drug-target interaction (DTI) data from two different sources: (1) DrugBank [54] and (2) from a previous DTI prediction study by Yamanishi et al. [55]. The DrugBank database freely provides high-quality curated data regarding drugs and drug-targets for conducting in silico bio and chemoinformatics studies. DrugBank DTI data was downloaded on October 4 2015 (version 4.3). All DTIs were conveniently represented as a list of pairs, along with protein sequence information for each target, and SMILE format for each drug. Any drug or drug target without a valid SMILE or protein sequence, respectively, was removed from our data set. The latter comprises DTI data from KEGG BRITE [56,57], BRENDA [58], SuperTarget [59] and DrugBank [54] from November 2007 and has been used as a gold standard in several DTI prediction studies [12,60–63]. Since Yamanishi’s data [55] contains positive instances from DrugBank, all duplicated entries between the two data sets were removed (Fig 3). In this study we disregarded the specific classes of protein targets (i.e., enzymes, G-protein coupled receptors, ion channels, and nuclear receptors) and excluded proteins with unreviewed status in the UniProt Knowledgebase [64] (i.e., proteins automatically annotated in TrEMBL). The number of unique drugs in our positive data sets is 2,118, comprising 1,328 from DrugBank and 790 from Yamanishi’s data [55]. In the same data, the number of unique drug-targets is 2,077 (706 from DrugBank and 1,371 from Yamanishi [55]). Finally, the number of known DTIs between the drugs and targets in the positive data is 10,736 (3,530 from DrugBank and 7,206 from Yamanishi [55]).

### Negative data set construction

Ideally, the negative data set should also exclusively comprise experimentally determined non-DTIs. However, very few authors publish non-interacting protein data, as these are generally associated with failed hypothesis. To collect experimental negative data we screened the BindingDB [65] and BioLip [66] databases for DTI pairs with experimental bioactivity values greater than 10 μM (Fig 3). The same strategy was previously applied to compile negative examples in another DTI prediction methodology [67], since DTIs with bioactivity values above this threshold are considered to possess weak binding activity. BindingDB data was downloaded in December 2015, and BioLip data was downloaded in April 2016. The number of unique DTIs with experimental bioactivity values greater than 10 μM in BindingDB and BioLip is 14,985 and 1,223, respectively. In the former, the number of unique drugs and drug-targets is 12,454 and 404, respectively. The latter comprises 894 unique drugs and 636 unique drug-targets.

### Machine learning data set construction

To ensure the discriminative power of the proposed approach, we used OpenBabel [68] to extract the molecular fingerprints of the drugs in our data sets and to compare their chemical similarity. We have found that within each data set (DrugBank, Yamanishi [55], BindingDB and BioLip) and across all data sets, less than 1% of all possible drug pairs had a sequence similarity score greater than 0.85. Then, we combined the described positive and negative data to construct the data sets for classification model training and external validation (Fig 3). The negative data collected from BindingDB and BioLip was merged and duplicated instances were removed, resulting in 16,209 unique negative DTIs. Each instance of these data was randomly selected and appended to one of the positive data sets (first to the Yamanishi [55] data set and then to the DrugBank data set) until all instances were exhausted, while maintaining a similar negative to positive ratio (approximately 1.5). Whenever a negative instance was randomly selected from the negative data to be appended in either positive data set, that instance was removed from the negative data set to ensure the absence of duplicated entries. This resulted in 18,118 instances in the training data set, consisting of 7,206 positive instances from the Yamanishi [55] data and 10,912 randomly selected negative instances. The external validation data set comprised 3,530 positive examples from DrugBank data and 5,297 randomly selected negative examples, totaling 8,827 DTIs.

### Calculation of the bacterial interactome network metrics

The interactome of the methicillin-resistant *S*. *aureus* (strain COL–MRSA COL) was downloaded from the STRING database [69] in January 2016. The 300,477 protein-protein interactions (PPIs) downloaded included 36,230 unique proteins, comprised of 9,875 active proteins and 26,355 obsolete in UniProt. From the active proteins, only 1,074 were reviewed and manually annotated in Swiss-Prot [64]. To avoid the presence of false-positive proteins in our experiments, we only considered reviewed proteins in this study. As a result, the MRSA COL interactome filtered for reviewed proteins comprised 93,952 PPIs. To calculate the subgraph centrality (SC) and betweenness centrality (BC) of each protein in the MRSA COL interactome we used NetworkX (https://networkx.github.io/), a Python software package for the creation and study of complex networks.

The subgraph centrality (SC) metric can be calculated from the spectra of the adjacency matrix of the network and was found to be better at discriminating the nodes of a network than alternative measures (e.g., degree, closeness). In addition, it was shown that SC is more highly correlated with the lethality of individual proteins removed from the proteome, compared with the number of links per node [49,70]. For a given node *u* the SC is given by,

$$SC(u)=\sum_{j=1}^{N}{{(v}_{j}^{u})}^{2}e^{\lambda_{j}}$$ (2)

where *v*_*j*_ is an eigenvector of the adjacency matrix A, corresponding to the eigenvalue *λ*_*j*_ obtained from the graph.

Bottlenecks in protein networks can be predicted by calculating the betweenness centrality, BC, with greater values suggesting a higher “bottleneck-ness”. These are networks nodes that have many shortest paths passing through them, making them key connector proteins. In comparison with degree centrality (i.e., “hub-ness”), bottlenecks are significantly better associated with essentiality [50]. For a node *v*, the BC is given by,

$$BC(v)=\sum_{s,t\in V}\frac{\sigma (s,t|v)}{\sigma (s,t)}$$ (3)

where *V* is the set of nodes, the denominator is the number of shortest paths in the network, and the numerator the number of those that pass through v.

### Computation of drug and protein descriptors

Drug and protein descriptors were computed using PyDPI, a python package for chemogenomics studies [58]. PyDPI calculates the most frequently used structural and physicochemical properties of a protein given its amino acid sequence, molecular descriptors of a drug from its smile representation, protein-protein interaction (PPI) descriptors, and DTI descriptors.

Using PyDPI we calculated 755 descriptors for each DTI– 432 protein descriptors and 323 drug descriptors. The 432 protein descriptors are divided as follows: 20 amino acid composition descriptors; 240 Moran autocorrelation descriptors, and; 147 CTD (21 Composition, 21 Transition, and 105 Distribution) physicochemical descriptors. The amino acid composition group of descriptors represents the fraction of each amino acid type in the sequence. Autocorrelation descriptors express the level of correlation between two proteins regarding specific structural or physicochemical properties. The CTD descriptors group represent the amino acid distribution pattern of specific structural or physicochemical properties along the primary structure of a protein, including hydrophobicity, polarity, charge, polarizability, normalized van der Waals volume, secondary structures and solvent accessibility. Drug features comprise 30 molecular constitutional descriptors, 23 molecular connectivity indices, six molecular property descriptors, seven kappa shape descriptors, 12 charge descriptors, 166 Molecular Access System (MACCS) keys, and 79 E-state fingerprints. Constitutional descriptors characterize the chemical element and chemical bond type, path length, hydrogen bond and hydrogen acceptor, while molecular and valence connectivity are described with the connectivity indices. For instance, Kappa indices reflect shape attributes of the molecule, and charge descriptors express electronic features of the whole molecule and of particular regions (atoms, bonds, and molecular fragments). Molecular fingerprints encode chemical structures which consist of bins, with each bin being a substructure descriptor associated with a specific molecular feature. A detailed explanation of these and other descriptor groups is given in the original publication of the PyDPI package [58].

### Drug-target interaction classification

The predictive model used in this study was implemented using scikit-learn, a Python package to perform data mining, data analysis and machine learning tasks [71]. To predict DTIs we implemented a classification model based on random forests of decision trees (RF) [48], which has been shown [72–74] as the best approach to solve complex classification problems in large data sets with a significant number of features. A random forest is an ensemble of many classifiers of the same base type (e.g., decision trees) which returns the class that is the mode of the classes across the output of the individual trees in the forest [48]. Each tree is fully constructed from a bootstrap sample drawn from the training set, by recursively splitting an upstream node. When splitting a node in the tree, the chosen split is only the best split among a random subset of features to prevent correlation between trees. This results in a split that is not the best split among all features, adding some randomness to the model and slightly increasing the forest bias. However, due to averaging between trees, the variance of the forest will also decrease, more than compensating the increase in bias and resulting in an overall better model. The trees are grown until a node cannot be split further. Conversely to the original model [48] where each tree votes for a single class, prediction of the class of input samples in the scikit-learn implementation is performed by averaging their probabilistic predictions. The number of trees (*n_estimators*) and the number of features to consider when looking for the best split (*max_features*) are important parameters when building an RF model. To define these parameters we used the grid search method and then adopted the parameters of the model with best mean accuracy after five-fold cross-validation. Thus, the parameters used were 150 *n_estimators* and 100 *max_features*. Since we only consider 100 features at each split, we believe over-parameterization does not occur.

The pipeline for the construction of our classification model is very straightforward: (1) train the RF; (2) assess internal classifier performance by five-fold cross-validation; (3) classify the external validation data set to evaluate classifier performance on out-of-sampling data, and; (4) classify the test data.

### Predictive model validation

While a performance comparison with the method proposed by Cheng *et al*. [12] would be ideal to ascertain how our approach compares with the state-of-the-art, the links to their data sets are unavailable by the time of writing. Thus, to estimate the classification accuracy of the implemented predictive models we used internal and external validation. Internal validation was performed using five-fold cross validation, which consists of splitting the training set in five subsets, using four subsets to train the model, and testing on the remaining subset. This is done consecutively until every subset is used as the test set. External validation was performed by using a data set independent from the training data as test set for the classification model. This strategy is fundamental to better estimate the performance and generalizability of the classifier, as cross-validation estimates are usually biased towards over-performance [75,76].
